# Regional citrate anticoagulation versus no-anticoagulation for continuous venovenous hemofiltration in patients with liver failure and increased bleeding risk: A retrospective case-control study

**DOI:** 10.1371/journal.pone.0232516

**Published:** 2020-05-05

**Authors:** Yan Yu, Ming Bai, Feng Ma, Wei Zhang, Yangping Li, Lijuan Zhao, Li Li, Meilan Zhou, Lu Li, Shiren Sun

**Affiliations:** Department of Nephrology, Xijing Hospital, The Fourth Military Medical University, Shaanxi, China; Azienda Ospedaliero Universitaria Careggi, ITALY

## Abstract

**Objective:**

There are controversial opinions on anticoagulation for continuous venovenous hemofiltration (CVVH) in patients with liver failure (LF) and increased bleeding risk. Therefore, we conducted a retrospective study to evaluate the efficacy and safety of regional citrate anticoagulation (RCA) versus no-anticoagulation for CVVH in these patients.

**Methods:**

The included patients were divided into RCA and no-anticoagulation group according to the CVVH anticoagulation strategy they accepted for CVVH. Filter lifespan, bleeding, citrate accumulation, catheter occlusion, and totCa/ionCa ratio were evaluated as outcomes.

**Results:**

In the original cohort, the filter lifespan of the RCA group (41 patients, 79 filters) was significantly longer than the no-anticoagulation group (62 patients, 162 filters) (> 72 hours vs 39.5 hours (IQR 31.2–47.8), *P* = 0.002). The adjusted results demonstrated that RCA could significantly reduce the risk of filter failure (HR = 0.459, 95%CI 0.26–0.82, *P* = 0.008). Four episodes of totCa/ionCa > 2.5 were observed in the RCA group and continuously accepted RCA-CVVH after the reduction of citrate dose and blood flow. No obvious citrate accumulation was observed in these patients. In the matched cohort, the filter lifespan of the RCA group was significantly longer than the no-anticoagulation group (*P* = 0.013) as well. No significant difference in the episodes of totCa/ionCa > 2.5 was observed between the two matched groups (*P* = 0.074). Both in the original cohort and the matched cohort, the bleeding, acidosis, alkalosis, and catheter occlusion incidences were not significantly different between the two groups.

**Conclusions:**

In LF patients with increased bleeding risk who underwent CVVH, RCA could prolong the filter lifespan and be safely used with careful blood gas monitoring and citrate dose adjusting. Further prospective, randomized, control studies are warranted to obtain robust evidences.

## Introduction

Continuous venovenous hemofiltration (CVVH) is commonly used in critically ill patients for the management of acute kidney injury (AKI), severe metabolic disorder, and refractory fluid overload. AKI were observed in 40–85% of acute liver failure (LF) [1], 24% of liver cirrhosis [2], and 10–30% of liver transplantation patients [3, 4]. And, most of these patients needed CVVH treatment to replace the kidneys to clear the endogenous toxins and the excessive water and to balance the electrolyte and acid-base status. Additionally, according to the European Association for the Study of the Liver (EASL) guideline, CVVH should be early instituted for persistent hyperammonaemia, hyponatraemia, metabolic abnormalities, and temperature control in LF patients [4]. Therefore, in clinical practice, LF is a common co-morbidity in critically ill patients underwent CVVH.

During CVVH treatment, clotting in the extracorporeal circuit shortens the filter and catheter lifespan, causes blood loss, and decreases solute clearance, consequently, reduces the effectiveness of CVVH and increases the treatment cost and medical stuff’s workload. Our previous meta-analysis demonstrated that regional citrate anticoagulation (RCA) for continuous renal replacement therapy (CRRT) could prolong the filter lifespan and decrease the bleeding risk, compared with heparin anticoagulation [5]. However, most of the included trials excluded the patients with LF over the impaired coagulation and impaired metabolic ability of anticoagulants. The Kidney Disease Improving Global Outcomes (KDIGO) guideline listed severe LF as a major contra-indication to RCA [6]. Patients with LF are often associated with coagulation abnormalities, thrombocytopenia, portal hypertension, esophageal and gastric fundus venous hemorrhage, all of which indicated increased bleeding risk. The reported incidences of bleeding were 50–80% and 50–63% in acute liver failure patients and decompensated cirrhosis patients, respectively [7–9]. Accordingly, for patients with LF and increased bleeding risk, CVVH should be performed without anticoagulation [10]. However, in clinical practice, we observed that parts of LF patients underwent CVVH with no-anticoagulation would result in relatively shorter filter lifespan. Previous study reported that the mean CVVH circuit lifespan was 7–8 hours in the no-anticoagulation group in patients with LF and coagulopathy [11]. And, several studies suggested that the use of RCA in LF patients did not result in incremental adverse events and could extend the filter lifespan, which suggested potential benefit of RCA anticoagulation in LF patients, especially in LF patients with increased bleeding risk [12–15]. Our previous systematic review pooled 10 observational studies and demonstrated that the RCA might be safe and effective for LF patients underwent CRRT. We also found out that all of the current evidences were limited in observational cohort study and none of the included studies evaluated the safety and efficacy of RCA versus no-anticoagulation (strategy recommended by the KDIGO guideline) in LF patients with increased bleeding risk [16].

Therefore, the purpose of our present study is to assess the efficacy and safety of RCA-CVVH versus no-anticoagulation in LF patients with increased bleeding risk in a retrospective observational cohort study.

## Methods

### Patients

This was a retrospective cohort study from a single center with 133 ICU beds, and approximately 2000 critically ill patients accepted CVVH treatment in our center per year. Patients with LF and increased bleeding risk who received CVVH therapy in our center between January 2013 and October 2016 were considered as candidates. We excluded patients if they met any of the following criteria: younger than 18 years, patients underwent low molecular weight heparin (LMWH) anticoagulation, patients underwent heparin anticoagulation, patients with severe hyperlacticaemia. According to the anticoagulation strategy for CVVH, the included patients were divided into the RCA group and no-anticoagulation group. Additionally, the patients in the original group were matched at 1:1 ratio according to the type (acute/chronic) and severity of LF.

The study was approved by the Ethics Committee of Xijing Hospital, the First Affiliated Hospital of Fourth Military Medical University and performed in accordance with the Declaration of Helsinki. The ethic committee waived the need for informed consent because of the retrospective study design.

### Characteristics of the CVVH protocol

The initiation of CVVH was decided by the attending doctor based on the KDIGO and EASL guideline [4, 6]. Briefly, the indications for CVVH included metabolic disorders (azotemia, hypernatremia, hyperkalemia, severe acidosis, etc.), fluid overload, persistent hyperammonaemia, temperature control, and sepsis. Temporary vascular access was created by inserting a dual lumen catheter into the femoral vein or jugular vein. CVVH were performed by using the Prismaflex devicer with M100 Set system (Gambro, Sweden), which had an effective membrane area of 0.9 m^2^ or AV600S (Frensius, German) with membrane area of 1.2 m^2^. The effluent flow rate was set at the routine speed of 2 L/h for normal-weighted patient, and the dose was adjusted according to the KDIGO guideline for over-weighted patient (>100 kg). Filter was routinely replaced every 72 hours, even though it was still functional.

In the RCA group, the initial blood flow rate was 180 ml/min, and the dose of 4% sodium citrate was 200 ml/min to achieve the postfilter ionized calcium between 0.25–0.35 mmol/L. The systemic ionized calcium concentration was titrated to be maintained in 1.0–1.3 mmol/L by the supplement of 10% calcium gluconate solution. Intensive metabolic monitoring, including acid-base status, sodium, potassium, and total and ionized calcium levels, was performed at 2-hour after the CVVH treatment and every 4 hours thereafter. The dose of citrate and calcium gluconate was adjusted based on the results of the postfilter and systemic ionized calcium levels. The nomograms for citrate and calcium gluconate adjustment are showed in Supplementary S1 and S2 Tables [17]. On the condition of totCa/ionCa > 2.5, the dose of 4% sodium citrate and the blood flow rate were modified to avoid electrolyte or metabolic derangements.

In the no-anticoagulation group, the CVVH treatment was performed without the use of any anticoagulation. The initial blood flow of no-anticoagulation CVVH was 200 mL/min.

### Date collection

Data were retrieved from the electronic medical records of our hospital. Baseline characteristics (demographic, clinical, biochemical data and Child-Pugh score, CVVH treatment indications), the blood gas results, liver function test results, renal function results, electrolyte test results, fluid removal, and the treatment-related complications (filter failure, catheter occlusion, bleeding, and citrate accumulation) during the CVVH treatment, and hospital mortality were recorded.

### Endpoints and definitions

Filter lifespan was defined as the time from the beginning of CVVH treatment to the filter replacement or CVVH termination due to one of the following reasons: TMP (transmembrane pressure) ≥ 300 mmHg, extracorporeal coagulation due to blood clots, CVVH termination caused by non-clotting events (the achievement of treatment goal, severe hypotension, death, and the upper limited time for filter (72 hours). Safety was assessed by the frequency of adverse events defined as bleeding, catheter occlusion, totCa/ionCa > 2.5, acidosis (pH < 7.35), and alkalosis (pH > 7.45). Bleeding was defined as having definite site of gross bleeding and at least one of the following criteria: drop of mean arterial pressure 10 mmHg, transfusion (requiring ≧ 2 red blood cells units) within 24 h, decrease in haemoglobin of ≧ 20 g/L, failure of haemoglobin increase after RBC transfusion [18]. Metabolic acidosis with an increased anion gap, decreasing ionized calcium, elevated total calcium and the calcium ratio (totCa/ionCa) > 2.5 were considered as citrate accumulation [19, 20].

Acute LF was defined as mental alteration (encephalopathy), and coagulation abnormality, usually INR > 1.5 (no anticoagulant condition) in a patient without pre-existing liver disease and the duration of illness less than 26 weeks [21]. And, chronic LF was defined as decompensated liver cirrhosis mainly manifested by ascites, portal hypertension, coagulation dysfunction and hepatic encephalopathy. Patients with one of the following characteristics were considered to be with increased bleeding risk: platelet count below 40 × 10 ^9^/L, activated partial thromboplastin time (APTT) longer than 60 seconds, INR > 2.0, bleeding within 7 days or active bleeding, recent trauma or surgery (especially head trauma and neurosurgery), recent stroke, intracranial arteriovenous malformations or aneurysm, retinal hemorrhage, and uncontrolled hypertension [6, 22].

### Statistical analysis

Continuous variables were presented as means and standard deviations, and categorical variables were presented as numbers (%). We used Student-*t* test to assess the difference between groups for normal-distribution continuous variables and Mann-Whitney rank test for non-normal distribution continuous variables. χ^2^ test or Fisher’s exact test were employed to assess the difference between groups for categorical variables. Classified variables was presented as median (interquartile range) and compared with Mann-Whitney U test. Additionally, the paired *t*-test was employed for repeated measure variables. Filter lifespan was assessed by the Kaplan-Meier survival curve and log-rank test, and the risk factors of time-dependent outcomes were identified by Cox regression model. All tests were two-sided, and a *P* value < 0.05 was considered statistical significant. Statistical analysis was run by using SPSS 16.0.

## Results

### Study population

**Fig 1:**
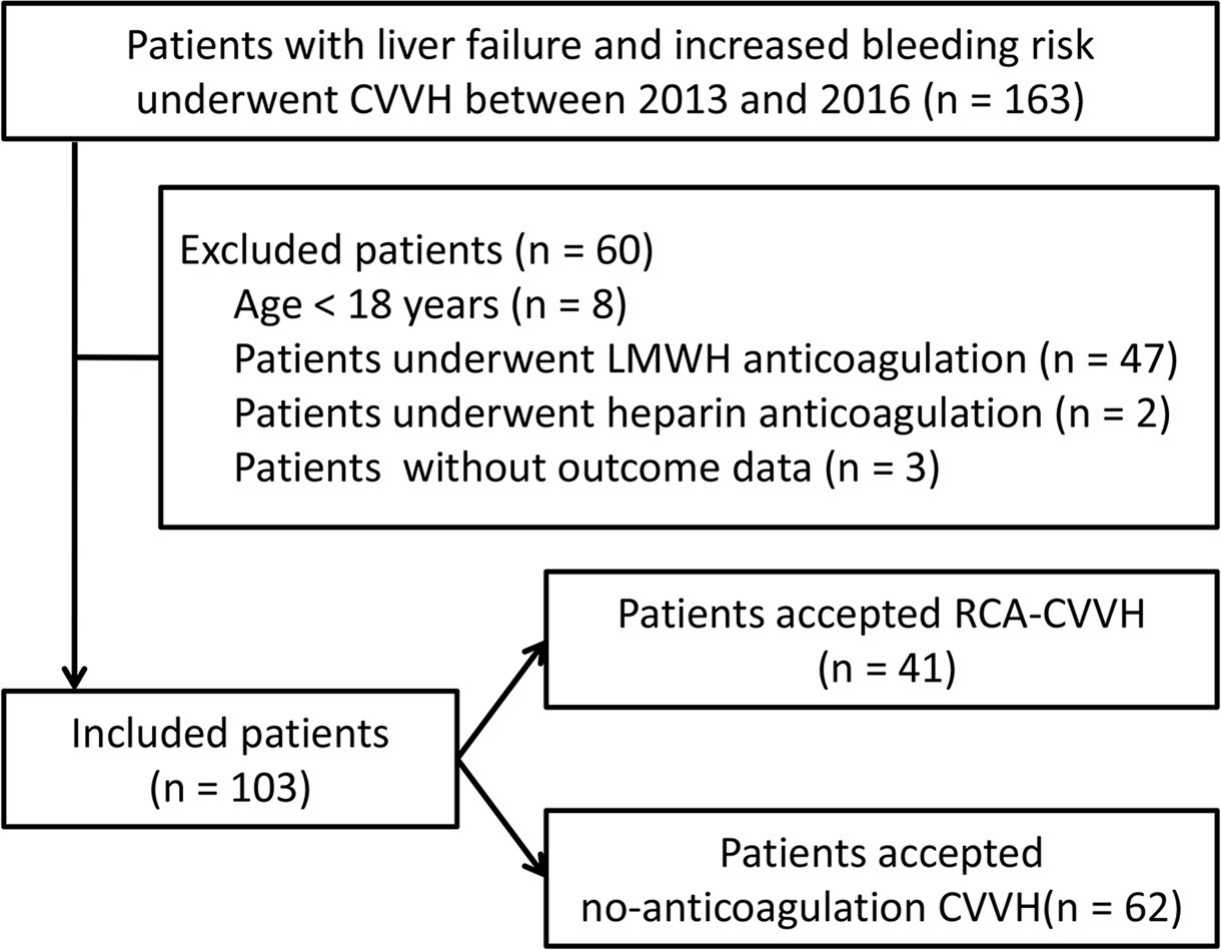
Patient inclusion flowchart.

Between January 2013 and January 2016, 163 consecutive ICU and Emergency Department patients with LF who received CVVH therapy were enrolled. Of these patients, 60 patients were excluded based on the exclusion criteria. At last, 103 patients were included in the original cohort. Before CVVH, there were 29 patients with active bleeding, 34 patients with recent (within 7 days) surgery, 5 patients with recent trauma, 3 patients with recent stroke, and 32 patients with coagulopathy and thrombocytopenia. Of the included patients, 41 patients underwent RCA CVVH with 79 filters and 62 patients underwent no-anticoagulation CVVH with 162 filters (Fig 1).

### Baseline characteristics

The baseline characteristics of the included patients are described in Table 1. In the original cohort, patients in the no-anticoagulation group had higher bilirubin (*P* < 0.001), APTT (*P =* 0.006) value, Child-Pugh score (*P* = 0.001), MELD score (*P* = 0.014), and AKI stage (*P* = 0.017) than the RCA group, but lower BUN levels (*P* = 0.045). The two groups were not significantly different in the remaining baseline characteristics (Table 1). Only part of patients (40/163, 38.8%) had the value of serum ammonia before and at the end of CVVH. There was no significant difference in the baseline serum ammonia between the RCA and no-anticoagulation group (136.84 ± 42.24 μmol/L vs. 149.5 ± 71.4 μmol/L, *P* = 0.741). The delivered dose was 34.8 ± 5.6 ml/kg/h in the RCA group and 30.9 ± 5.8 ml/kg/h in the no-anticoagulation group.

**Table 1:** Baseline characteristics of the included patients in the original groups.

Variables	RCA-CVVH (n = 41)	No-anticoagulation-CVVH (n = 62)	*P*-value
Gender, male, n (%)	22 (53.7)	42 (67.7)	0.149
Age, years	52.49 ± 15.62	47.13 ± 14.08	0.073
APACHE II	17.12 ± 4.30	16.00 ± 5.73	0.286
SOFA	11.44 ± 4.04	11.65 ± 4.35	0.809
Child-Pugh score	8.51 ± 1.31	9.37 ± 1.26	0.001
MELD score	29.78 ± 6.93	34.74 ± 11.32	0.014
Bilirubin, μmol/L	67.57 ± 26.10	184.95 ± 138.38	<0.001
PLT, 10^9^/L	98.8 ± 60.65	90.19 ± 70.97	0.525
FIB, g/L	2.29 ± 1.36	1.67 ± 1.16	0.050
APTT, s	45.86 ± 29.52	66.34 ± 36.56	0.006
PTA, %	30.67 ± 17.41	28.84 ± 16.21	0.588
INR	2.35 ± 1.27	2.67 ± 1.48	0.261
BUN, mmol/L	23.57 ± 13.57	18.44 ± 11.85	0.045
Serum Creatinine, μmol/L	297.83 ± 141.28	324.56 ± 251.37	0.537
Liver failure, acute, n (%)	39 (95.1)	51 (82.3)	0.054
AKI stage, 1/2/3, n (%)	0(0)/13(31.7)/28(68.3)	5(8.1)/11(17.7)/43(69.4)	0.017
Hypernatremia, yes, n (%)	4 (9.8)	3 (4.8)	0.432
Hyperkalemia, yes, n (%)	15 (36.6)	17 (27.4)	0.325
Fluid overload, yes, n (%)	12 (29.3)	10 (16.1)	0.111
Sepsis, yes, n (%)	4 (9.8)	7 (11.3)	1.000

RCA, Regional citrate anticoagulation; CVVH, continuous venovenous hemofiltration; PLT, platelet counts; FIB, fibrinogen; APTT, activated partial thromboplastin time; PTA, prothrombin time activity; INR, international normalized ratio; BUN, blood urea nitrogen; AKI, Acute kidney injury. Unless indicated otherwise, data are presented as the mean ± standard deviation (SD).

### Efficacy outcomes

**Fig 2:**
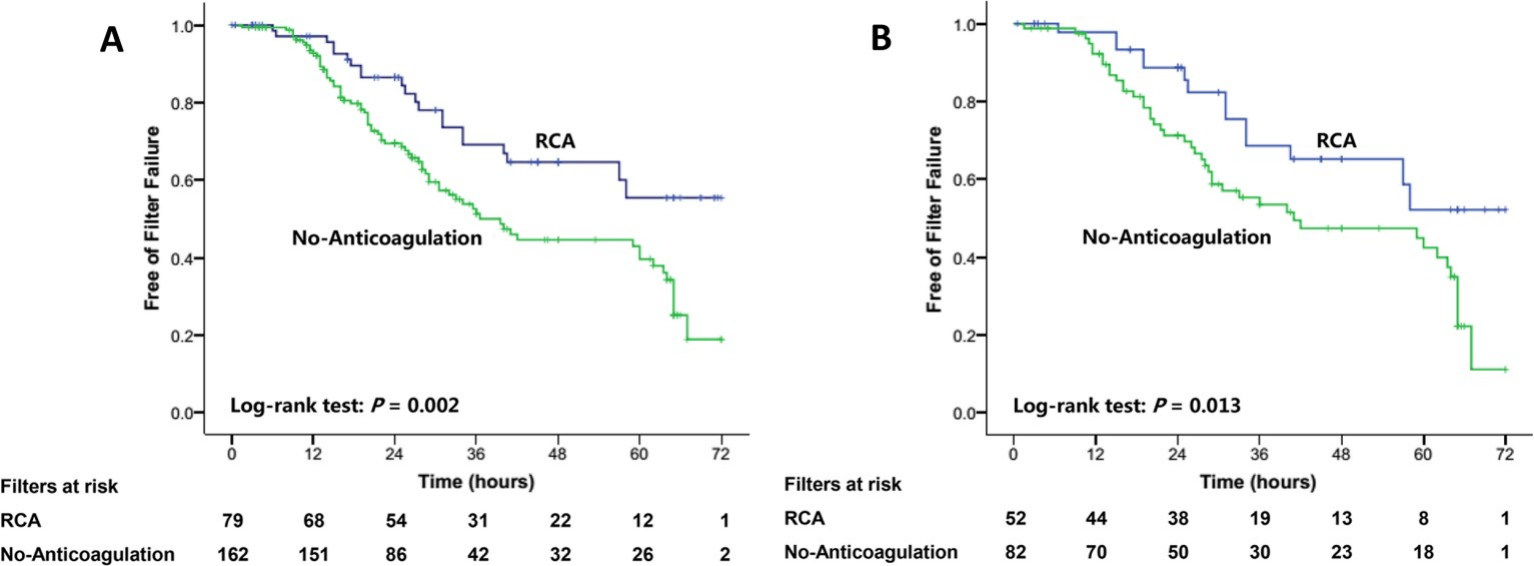
Survival curves of the filters with the use of RCA versus the filters with the use of no-anticoagulation in the original cohort (A) and the matched cohort (B).

The estimated median filter lifespan was longer than 72 hours in the RCA group and 39.5 hours (IQR 31.2–47.8) in the no-anticoagulation group. The RCA group had significantly longer estimated median filter lifespan compared with the no-anticoagulation group (*P* = 0.002, Fig 2A). The accumulated 24-, 48-, and 72-hour filter failure rates were 13.5%, 35.3%, and 44.6% in the RCA group and 30.5%, 55.4%, and 81.1% in the no-anticoagulation group, respectively.

In univariate analyses, RCA (*P* = 0.003), PLT (*P* = 0.008), and APTT (*P* = 0.045) were significantly related to filter lifespan. In the multivariate COX regression model, the risk of filter failure was significantly reduced by RCA (*P* = 0.001, HR = 0.392) after the correction of the two factors (PLT and APTT) identified in univariate analysis. Moreover, after adjusting the clinically significant indicators (filter type, vascular access, PLT, and INR), RCA significantly reduced the risk of filter failure (HR = 0.459, 95% CI 0.26–0.82, *P* = 0.008) as well. Other identified risk factors of filter failure included HBG (HR = 1.009, 95% CI 1.001–1.018, *P* = 0.036) and APTT (HR = 1.008, 95% CI 1.001–1.015, *P* = 0.018, Table 2).

**Table 2:** Predictors of filter failure in CVVH patients with liver failure and increased bleeding risk.

		Univariate Cox regression			Multivariate Cox regression			Multivariate Cox regression adjusted the important clinical parameters		
		HR	95%CI	*P* value	HR	95%CI	*P* value	HR	95%CI	*P* value
Anticoagulation strategy (RAC/no-anticoagulation)		0.48	0.295–0.779	0.003	0.392	0.222–0.695	0.001	0.459	0.259–0.815	0.008
Filter Type (M100 / AV600)		0.578	0.180–1.856	0.357	-	-	-	109626.395	0.000	0.974
Vascular access		0.325	0.079–1.337	0.119	-	-	-	0.384	0.092–1.604	0.19
PLT		0.994	0.990–0.999	0.008	0.997	0.993–1.001	0.182	0.996	0.992–1.001	0.99
APTT		1.005	1.000–1.010	0.045	1.007	1.002–1.012	0.004	1.008	1.001–1.015	0.018
INR		1.001	0.999–1.003	0.284	-	-	-	1.010	0.822–1.242	0.922
HBG		1.003	0.996–1.010	0.34	-	-	-	1.009	1.001–1.018	0.036

RCA, Regional citrate anticoagulation; CVVH, continuous venovenous hemofiltration; PLT, platelet; APTT, activated partial thromboplastin time; INR, international normalized ratio; HBG, hemoglobin.

### Safety outcomes

Seven (7/162, 4.3%) and 4 (4/79, 5.1%) bleeding episodes occurred in the no-anticoagulation and RCA group (*P* = 0.753), respectively. The two groups were not different in the units of red blood cell transfusion required during CVVH (6.7 ± 5.5 vs. 3.9 ± 3.5, *P* = 0.318, Table 3) and site of bleeding. Of the included patients, 3 in the RCA group (3/62, 4.8%) and 2 patients (2/41, 4.9%) in the no-anticoagulation group accepted vitamin K1 injection during their CVVH treatment. The two groups were not different in the use of vitamin K injection. None was given clotting factors during their CVVH treatment. There was no difference in the episodes of catheter occlusion (0.6% vs. 2.5%, *P* = 0.251) and acidosis (5.0% vs. 9.1%, *P* = 0.226) between the two groups. In addition, no central venous thrombosis or thromboembolism events were observed in our present cohort. No alkalosis (pH > 7.45) was observed in both the two groups.

**Table 3:** Adverse events among the liver failure and increased bleeding risk patients in original and matched cohort.

Endpoints	Original cohort			Matched cohort		
	RCA-CVVH	No-anticoagulation-CVVH	*P*-value	RCA-CVVH	No-anticoagulation-CVVH	*P*-value
Bleeding, n (%)	4 (5.1)	7 (4.3)	0.753	3 (5.8)	4 (4.9)	1.000
RBC Transfusion (units), mean ± SD	6.7 ± 5.5	3.9 ± 3.5	0.318	7.6 ± 6.3	5.9 ± 3.8	0.656
Catheter occlusion, n (%)	2 (2.5)	1 (0.6)	0.251	1 (1.9)	0 (0)	1.000
TotCa/ionCa > 2.5, n (%)	4 (5.1)	0 (0)	0.004	2 (3.8)	0 (0)	0.074
Acidosis, pH < 7.35, n (%)	7 (9.1)	8 (5.0)	0.226	5(9.6)	5 (6.1)	0.450
Alkalosis, pH > 7.45, n (%)	0 (0)	0 (0)	0.237	0 (0)	0 (0)	0.134

RCA, regional citrate anticoagulation; CVVH, continuous venovenous hemofiltration; TotCa/ionCa, total to ionized calcium ratio.

The incidences of totCa/ionCa > 2.5 was higher in the RCA group (4 out of 79 CVVH runs) compared with the no-anticoagulation group (0 out of 162 CVVH runs, Table 3). All of the patients did not have citrate accumulation during their CVVH treatment after the reduction of the sodium citrate dose and blood flow and the increase of the calcium supplementary.

### Other outcomes

At the end of CVVH, there was no significant difference in the serum urea (13.04 ± 6.90 mmol/L vs. 11.53 ± 7.14 mmol/L, *P* = 0.340), and ammonia (72.15 ± 39.39 μmol/L vs. 79.43 ± 32.89 μmol/L, *P* = 0.785) between the RCA group and no-anticoagulation group. Net ultrafiltrate flow rate during CVVH was 43.8 mL/hr [IQR, 0–52.7] in the RCA group and 43.1 mL/hr [IQR, 0–69.8] in the no-anticoagulation group (*P* = 0.783).

The averaged follow-up time was 12.8 ± 9.6 days. During the hospital staying, 2 patients in the RCA group and one patient in the no-anticoagulation group underwent plasma exchange. Of the included patients, no patients underwent MARS. There was no difference in the routine aspects of care between the two groups. The in-hospital mortality was not significantly different between the two groups (RCA: 63.4%, n = 26 vs. no-anticoagulation: 54.8%, n = 34; *p* = 0.421). One patient in the RCA group accepted liver transplantation at three months and one patient in the no-anticoagulation group accepted liver transplantation at eight months after the initiation of CRRT.

### Outcomes of the matched cohort

According to the course of LF (acute/chronic) and the degree of LF, 26 pairs of patients were matched. After the 1:1 matching, the two groups were not significantly different in the baseline characteristics (Table 4).

**Table 4:** Baseline characteristics of the included patients in the matched groups.

Variables	RCA-CVVH (n = 26)	No-anticoagulation-CVVH (n = 26)	*P*-value
Gender, male, n (%)	13 (50)	16 (61.5)	0.402
Age, years	51.19 ± 15.92	46.88 ± 14.61	0.314
APACHE II	17.31 ± 4.59	16.46 ± 4.90	0.524
SOFA	12.58 ± 4.02	11.38 ± 5.01	0.349
Child-Pugh score	8.58 ± 1.24	8.92 ± 1.02	0.833
MELD score	31.85 ± 7.16	32.23 ± 5.89	0.276
Bilirubin, μmol/L	78.61 ± 25.94	88.82 ± 50.47	0.363
PTA, %	27.67 ± 16.32	25.64 ± 13.32	0.626
INR	2.46 ± 1.52	2.66 ± 1.17	0.604
Serum Creatinine, μmol/L	350.08 ± 150.87	313.27 ± 149.71	0.381
Liver failure, acute, n (%)	26 (100)	26 (100)	1.000
AKI stage, 1/2/3, n (%)	0(0)/5(19.2)/21(80.8)	2(7.7)/5(19.2)/19(73.1)	0.238
Hypernatremia, yes, n (%)	1 (3.8)	0 (0)	1.000
Hyperkalemia, yes, n (%)	10 (38.5)	6 (23.1)	0.229
Fluid overload, yes, n (%)	8 (30.8)	4 (15.4)	0.188
Sepsis, yes, n (%)	4 (15.4)	3 (11.5)	1.000

RCA, Regional citrate anticoagulation; CVVH, continuous venovenous hemofiltration; PTA, prothrombin time activity; INR, international normalized ratio; AKI, Acute kidney injury. Unless indicated otherwise, data are presented as the mean ± standard deviation (SD)

In the matched cohort, 82 and 52 filters were used in the no-anticoagulation and RCA group, respectively. The estimated median filter lifespan was significantly longer in the RCA group (> 72 hours versus 41 (IQR 10.2–71.7) hours, *P* = 0.013, Fig 2B) compared with the no-anticoagulation group. The 24-hour accumulated filter failure proportion of the RCA group and the no-anticoagulation group was 11.3% and 28.7%, respectively.

Additionally, the incidences of total Ca^2+^/iCa^2+^ > 2.5 (3.8% vs. 0%), bleeding (5.8% vs. 4.9%), units of red blood cell transfusions required during CVVH (7.6 ± 6.3 vs. 5.9 ± 3.8), acidosis (9.6% vs. 6.1%), and catheter occlusion (1.9% vs. 0%) were not significantly different between the two groups. No alkalosis (pH > 7.45) was observed in both the two groups (Table 3).

## Discussion

To the best of our knowledge, our present study is the first cohort study evaluated the efficacy and safety of regional citrate anticoagulation (RCA) versus no-anticoagulation for CVVH in patients with LF and increased bleeding risk. Our present study has several findings. Firstly, the use of RCA for CVVH could significantly increase the filter lifespan in LF patients with increased bleeding risk, compared with the use of no-anticoagulation. Secondly, the use of RCA for CVVH did not significantly increase the risk of citrate accumulation, bleeding, metabolic acidosis, and alkalosis in LF patients with increased bleeding risk.

LF is a common co-morbidity of ICU patients who need CVVH treatment. And, most of LF patients are associated with increased bleeding risk. Currently, no-anticoagulation was recommended for CVVH in patients with severe LF and increased bleeding risk regarding the impaired citrate metabolic ability and contraindication to systemic anticoagulation. Usually, LF patients were considered to be associated with coagulopathy which could lead to extended CVVH filters. However, studies proved that LF patients were insufficient in both procoagulant (including von Willebrand factor and factor VIII) and anticoagulant factors (including thrombomodulin) leaded to the co-exist of hypo- and hypercoagulable situation [4, 23, 24]. These coagulation disorders most likely contributed to the shorter circuit lifespan for no-anticoagulation CVVH in LF patients with coagulopathy reported by Chua HR *et al*. (7.4–12 hours) and Banwari *et al* (10.5 hours) [11, 25]. In our present study, the 24-hour cumulative filter failure proportion was 30.5%. Commonly, a CVVH treatment course was at least 24 hours. Therefore, almost one third of the LF patients underwent no-anticoagulation CVVH needed filter exchange per treatment course, which directly lead to frequent filter exchange, increased down-time, decreased solute clearance, and higher treatment costs [26–28].

Several observational studies reported that the averaged circuit lifespan in LF patients underwent RCA-CVVH was 71.1 hours [12], 62.4 hours [29], and 72 ± 22.2 hours [30]. In our original and matched cohort, the estimated median filter lifespan was more than 24-hour and the accumulated proportions of filter failure were 13.5% and 11.3%, respectively. Compared with the patients in the no-anticoagulation group, the filter lifespan was significantly extended and the filter failure incidence was significantly reduced. Additionally, both the multivariate Cox regression model adjusted for the significant indicators in the univariate analysis and the model adjusted for the clinically important indicators identified RCA as one of the independent protective factors of filter failure. And, the HR value suggested that the use of RCA for CVVH could reduce more than 50% filter failure risk compared with no-anticoagulation CVVH in LF patients with increased bleeding risk.

Regarding the reduced citrate metabolic ability in LF patients which leaded to potential increased citrate accumulation risk, severe LF was considered as one of the contraindications for the use of RCA for CVVH [31, 32]. Currently, the blood citrate concentration was not routinely tested in most hospitals. The totCa/ionCa ratio > 2.5 has been commonly used as an indicator of citrate accumulation [13, 33, 34]. In the matched cohort, the incidence of totCa/ionCa > 2.5 in the RCA group was 3.8%. Several cohort studies have reported the use of RCA in patients with LF underwent CVVH and reported the incidences of citrate accumulation. In the study by Slowinski *et al*. [12], Sponholz *et al*. [35], and Lahmer *et al*. [36], the incidences of citrate accumulation were reported to be 2.2%, 3%, and 4%, respectively. And, no citrate accumulation was observed in the study by Durao *et al*. and De Vico *et al*.[14, 30]. However, Schultheiß *et al*. reported that the incidence of citrate accumulation was 16% in their cohort [13]. Most likely, these heterogeneity mainly caused by the differences in the severity of LF and RCA strategy, and especially the citrate accumulation definition. Khadzhynov D *et al*. considered that the increase of totCa/ionCa could be induced by other causes and was not specific enough for the diagnosis of citrate accumulation [20]. Therefore, they recommended more rigorous diagnosis criteria for citrate accumulation: (i) decreased systemic ionized calcium; (ii) increased demand for calcium substitution; (iii) elevated totCa/ionCa ratio; and, (iv) metabolic acidosis with or without an increased anion gap [19, 20].We employed this definition in our clinical practice and our present study.

Generally, during RCA-CVVH treatment, 40–60% of the sodium citrate given before the filter could be removed by the filter [37, 38]. The remaining citrate enter the systemic circulation and is metabolized in the liver, muscle and kidney through the tricarboxylic acid cycle [39]. The occurrence of citrate accumulation could lead to the reduction of ionCa. Patients with severe lower ionCa concentration might present tremor, convulsions, and severe arrhythmias [39]. These potentially serious adverse events could be immediately reversed by the increase of calcium infusion [40]. In our cohort, all of the patients with totCa/ionCa > 2.5 continually underwent RCA after the adjustment of the sodium citrate dose, blood flow, and calcium supplement infusion rate. And, no obvious citrate accumulation and symptom related to lower ionCa were observed. Possibly, in most of the LF patients, the remaining liver citrate metabolism ability and the citrate metabolism ability of other organs could fulfill the clearance of the system citrate to avoid citrate accumulation in RCA-CVVH [41].

Additionally, other outcomes including bleeding, alkalosis, metabolic acidosis, and catheter occlusion were commonly used to evaluate the safety of anticoagulation for CVVH [42]. No difference was observed between the two groups in these outcomes in both the original and matched cohort. Therefore, in the condition of closely monitoring and carefully dose adjusting, RCA most likely could be safely used in LF patients at high risk of bleeding.

Our present study has several limitations. At first, the retrospective nature should be considered as the major limitation. Some important baseline covariates were not distributed equally in the original cohort, which may bias the results. In order to reduce the influence of this imbalance, we matched patients according to the type (acute/chronic) and severity of LF and confirmed the results in the matched cohort. Secondly, the blood citrate concentration, liver function, and coagulation indicators were not routinely tested during CVVH treatment. Therefore, we have not reported the results of these outcomes, which should be evaluated in further prospective studies. Accordingly, we are performing a randomized controlled trial (NCT 03791190) to verify the findings of our present study and to present higher quality evidences for clinicians to choose appropriate anticoagulation for CVVH in LF patients.

## Conclusions

In conclusion, RCA for CVVH could significantly extend the filter lifespan in patients with LF and increased bleeding risk and may not significantly increase the risk of citrate accumulation, bleeding, catheter occlusion, metabolic acidosis, and metabolism alkalosis, compared with no-anticoagulation. Therefore, with intensive monitoring and careful dose adjusting, RCA might be safe and effective in patients with LF and increased bleeding risk who underwent CVVH. Further prospective, well designed, randomized controlled trial studies are warranted to provide higher quality evidences on this field.

## Supporting information

S1 Table Algorithm for adjustment of citrate dose. (DOCX)

S2 Table Algorithm for the adjustment of calcium dose. (DOCX)
