# Supplementary material for: Regional citrate anticoagulation versus no-anticoagulation for continuous venovenous hemofiltration in patients with liver failure and increased bleeding risk: A retrospective case-control study
Source: PLoS One. 2020 May 5;15(5):e0232516. doi: 10.1371/journal.pone.0232516 (PMC7199954; doi:10.1371/journal.pone.0232516)
Supplement: S1 Table — (DOCX) [file pone.0232516.s001.docx]

| Postfilter Ionized Ca（mmol/L） | Modification of Citrate Dose  (ml/h) | Recheck Postfilter Ionized Ca（mmol/L） |
| --- | --- | --- |
| <0.25 | ↓20 | 1 hours |
| 0.25-0.35 | No change | 4 hours |
| 0.36-0.40 | ↑10 | 1 hours |
| 0.41-0.45 | ↑20 | 1 hours |
| >0.45 | ↑30 | 1 hours |

**Supplementary Table 1. Algorithm for Adjustment of Citrate Dose**
