# Supplementary material for: Regional citrate anticoagulation versus no-anticoagulation for continuous venovenous hemofiltration in patients with liver failure and increased bleeding risk: A retrospective case-control study
Source: PLoS One. 2020 May 5;15(5):e0232516. doi: 10.1371/journal.pone.0232516 (PMC7199954; doi:10.1371/journal.pone.0232516)
Supplement: S2 Table — (DOCX) [file pone.0232516.s002.docx]

| Systemic Ionized Ca（mmol/L） | Modification of Calcium Dose | Recheck Systemic Ionized Ca |
| --- | --- | --- |
| >1.35 | ↓0.5 mmol/h | 4 hours |
| 1.0-1.35 | No change | 6 hours |
| 0.91-0.99 | ↑0.5 mmol/h | 6 hours |
| 0.86-0.90 | ↑1.1 mmol/h | 4 hours |
| 0.75-0.85 | ↑1.65 mmol/h | 2 hours |
| <0.75 | ↑2.2 mmol/h | 2 hours |

**Supplementary Table 2. Algorithm for the Adjustment of Calcium Dose.**
